# YOLOv8-Scm: an improved model for citrus fruit sunburn identification and classification in complex natural scenes

**DOI:** 10.3389/fpls.2025.1591989

**Published:** 2025-07-07

**Authors:** Guoxun Cong, Xinghong Chen, Zongyu Bing, Wenhuan Liu, Xiangling Chen, Qun Wu, Zheng Guo, Yongqiang Zheng

**Affiliations:** ^1^ National Digital Planting (Citrus) Innovation Sub-Center, National Engineering Research Center for Citrus Technology, Citrus Research Institute, Southwest University, Chongqing, China; ^2^ Citrus Research Institute, Southwest University, Chongqing, China; ^3^ Horticultural Research Institute, Guangxi Academy of Agricultural Sciences, Nanning, Guangxi, China; ^4^ Quzhou Academy of Agricultural and Forestry Sciences, Quzhou, Zhejiang, China

**Keywords:** YOLO v8n, YOLOv8-Scm, citrus sunburn, smart orchard monitoring, object detection

## Abstract

Citrus ranks among the most widely cultivated and economically vital fruit crops globally, with southern China being a major production area. In recent years, global warming has intensified extreme weather events, such as prolonged high temperature and strong solar radiation, posing increasing risks to citrus production,leading to significant economic losses. Existing identification methods struggle with accuracy and generalization in complex environments, limiting their real-time application. This study presents an improved, lightweight citrus sunburn recognition model, YOLOv8-Scm, based on the YOLOv8n architecture. Three key enhancements are introduced: (1) DSConv module replaces the standard convolution for a more efficient and lightweight design, (2) Global Attention Mechanism (GAM) improves feature extraction for multi-scale and occluded targets, and (3) EIoU loss function enhances detection precision and generalization. The YOLOv8-Scm model achieves improvements of 2.0% in mAP50 and 1.5% in Precision over the original YOLOv8n, with only a slight increase in computational parameters (0.182M). The model’s Recall rate decreases minimally by 0.01%. Compared to other models like SSD, Faster R-CNN, YOLOv5n, YOLOv7-tiny, YOLOv8n, and YOLOv10n, YOLOv8-Scm outperforms in mAP50, Precision, and Recall, and is significantly more efficient in terms of computational parameters. Specifically, the model achieves a mAP50 of 92.7%, a Precision of 86.6%, and a Recall of 87.2%. These results validate the model’s superior capability in accurately detecting citrus sunburn across diverse and challenging natural scenarios. YOLOv8-Scm enables accurate, real-time citrus sunburn monitoring, providing strong technical support for smart orchard management and practical deployment.

## 1 Introduction

Citrus is the most widely cultivated commercial fruit crop globally, with the highest yield among fruit species (Chen et al., 2017). In recent years, due to global warming, the frequency and severity of extreme weather events have increased (Khumalo and Ngcamphalala, 2024). During summer and autumn, citrus fruits exposed to intense sunlight and high temperatures may suffer from sunburn damage (Zhang et al., 2024). This sunburn damages the epidermal tissue of the fruit, making the skin rough and thickened, and halting fruit development during the expansion phase (Kim et al., 2022). This phenomenon, known as citrus sunburn, reduces fruit yield and quality (Mohsen and Ibrahim, 2021; Fischer et al., 2022). In agricultural practice, the rapid identification and classification of citrus sunburn are of great practical significance. It allows for the timely implementation of protective measures to reduce agricultural losses and is beneficial for their application in agricultural insurance disaster statistics (Campbell et al., 2004; Tarancón et al., 2021).

Currently, there are four main methods for determining the degree of citrus sunburn damage. Firstly, human judgment by agricultural technicians or citrus growers. However, this method is highly subjective and inefficient (Molin et al., 2012; Londhe et al., 2013; Naruka and Bhadauria, 2020). Secondly, using spectroscopic equipment to detect fruit (Gutiérrez et al., 2019; Moriya et al., 2021; Liu et al., 2024). Although it can monitor tissue damage both on and beneath the epidermis, the equipment is expensive (Xiu et al., 2021) and involves complicated procedures. Thirdly, conducting biological (Reetika et al., 2024) and molecular experiments (Zhang et al., 2020). This includes two aspects: (1) Anatomical observation and physiological experiments on the abiotic stress response. The results show that with the intensification of citrus sunburn, the fruit’s epidermal pigment increases, and the epidermal tissue and oil glands are damaged (Kim et al., 2022). (2) Determining proline and MDA (Malondialdehyde) (Vives-Peris et al., 2024), hydrogen peroxide and antioxidant enzyme activities, as well as chlorophyll analysis and phytohormone quantification (such as abscisic acid, salicylic acid, and auxins) under high-temperature stress (Balfagón et al., 2023) and UV-B-induced stress. However, this method can only determine changes in physiological indicators and the tolerance response of citrus fruits subjected to sunburn damage (Yun et al., 2013). Fourthly, machine vision-based fruit identification (Mirhaji et al., 2021; Lyu et al., 2022) and maturity grading (Wang et al., 2024). This is an emerging technique. Although machine vision methods have shown promising results, their precision significantly declines when dealing with small, occluded targets and complex natural environments (Gao et al., 2022; Zhao et al., 2024). However, the identification and grading method based on machine vision can be an accurate and rapid means to assess the fruits of a citrus sunburn disaster.

As deep learning and image processing technologies advance, machine vision has found increasingly widespread applications in agriculture (Ghazal et al., 2024; Padhiary and Kumar, 2024). One of its applications in agriculture is object recognition, which is frequently used to identify sunburn-related damage in fruits such as pomegranates (Rezaei et al., 2018), apples (Amogi et al., 2022), cranberries (Akiva et al., 2021), and citrus (Dhiman, 2022). Rezaei et al (Rezaei. et al., 2024). used the ANN (Artificial Neural Network) and SVM (Support Vector Machine) methods to automatically classify sunburned pomegranate fruits based on features extracted by the algorithm. However, these two methods mainly focus on classification tasks. Although they show high efficiency, their performance in multi-object detection tasks are suboptimal. Akiva et al (Akiva et al., 2021). proposed the Triple-S Network model. This model uses machine vision technology to segment and count the cranberry damaged part caused by thermal radiation. It has a mean absolute error (MAE) of 13.46 and a MIoU (mean intersection over union) of 62.54%. Although this study achieved the expected goals, the model’s accuracy and detection efficiency still have significant potential for further enhancement. Amogi et al (Amogi et al., 2023). trained the Mask R-CNN model and combined it with the color-based K-means clustering method to evaluate sunburned apples. The average precision was 91.4%, and the calculation time was 37s, which improved the monitoring speed. However, this model consumes a large amount of computing resources and has strict requirements for device hardware. Although it has high detection precision, its processing efficiency is low, and it is challenging to be light-weightly deployed on low-resource-overhead devices. The AG-YOLO algorithm was proposed to solve the problems of low detection precision and a high missed detection Rate in identifying citrus fruit (Lin et al., 2024). In occlusion scenes, it can provide fast and accurate technical means for citrus yield estimation (Zhang et al., 2024a). However, the purpose of these experiments was only to improve the identification and monitoring abilities of citrus fruits in complex environments (Xu et al., 2023; Zhang et al., 2024b) and the feature acquisition of the overall unit of citrus fruits, without classifying the local characteristics of the fruits. Mirhaji et al (Mirhaji et al., 2021). used the YOLO model for fruit detection and yield in citrus orchards via a simple method under image and lighting conditions. The precision rate, recall rate, F1 score and mAP of the model were 91.23%, 92.8%, 92% and 90.8%, respectively. This study was able to detect and identify fruits under different lighting environments but did not divide the grades and categories of navel citrus, and the model was unable to acquire specific differences in fruit surface characteristics. To address the deficiencies in previous studies, we conducted a study on the classification and identification of citrus sunburn fruits based on the YOLO algorithm (Jocher et al., 2023) and set five different labels. YOLO (You Only Look Once) is an efficient and precise algorithm for object detection. It converts the task of object detection into a single inference problem and utilizes a unified neural network to predict both the locations and categories of objects within an image simultaneously. The focus of this study was on the size, proportion and severity of the sunburned area of the fruit epidermis, classifying sunburned fruits into five levels (0-4) to obtain more detailed information on the outer surface characteristics of the fruit skin.

To address the above-mentioned issues, we propose a sunburn citrus level identification model based on an improved YOLO v8n algorithm. This model centers on the relationship between the surface damage degree of citrus fruits and the severity of citrus sunburn. Given the complex environmental conditions, the model mainly extracts the sunburn damage characteristics on citrus fruit surfaces. It detects citrus fruits at different scales, extracts the surface features of citrus fruits of different grades, and develops a high-precision, lightweight machine vision model (Lu et al., 2023). In particular, the identification of phenotypic traits related to different levels of sunburn provides faster and more accurate technical means for disaster assessment and statistics in citrus orchards. We enhanced the baseline YOLOv8n architecture by introducing three key modules:

Specifically: (1) The DSConv module replaces standard convolution to significantly reduce computational load while maintaining feature extraction capacity, thereby enabling the model’s deployment on lightweight agricultural devices. (2) The Global Attention Mechanism (GAM) is introduced to enhance the model’s sensitivity to sunburn-affected regions by capturing both global and spatial contextual cues, improving robustness against occlusion, background clutter, and target size variability. (3) The EIoU loss function refines bounding box regression by optimizing aspect ratio alignment and convergence speed, addressing the issue of inaccurate localization caused by scale and shape variations in citrus fruits. Collectively, these modules not only improve the detection precision and generalization ability of the model but also ensure its applicability in real-time, resource-constrained agricultural scenarios.

The primary objective of this study is to achieve a practical trade-off between classification precision and computational efficiency, enabling real-time inference and potential deployment on mobile or embedded agricultural platforms. While this work explores the deployment potential of YOLOv8-Scm on edge devices and panoramic orchard cameras, we emphasize that such deployment scenarios remain hypothetical at this stage. The model is expected to be deployed in practical production applications in the near future. Nevertheless, the deployment considerations are grounded in real-world demands for in-orchard monitoring, serving as a foundation for future engineering efforts toward lightweight, mobile-compatible agricultural stress detection tools.

The subsequent sections of this paper are arranged as follows. Section 1 centers on image acquisition, sample division, and dataset preparation. In Section 2, the baseline model is selected via preliminary experiments, and the architecture of the YOLOv8-Scm model along with the introduction of each module is presented. Section 3 presents the comparison results of the model before and after improvement and analyzes the model by means of ablation experiments and comparison tests. Section 4 discusses the limitations of the current study, proposes future research directions, and summarizes the main conclusions.

## 2 Materials and methods

**Figure 1:**
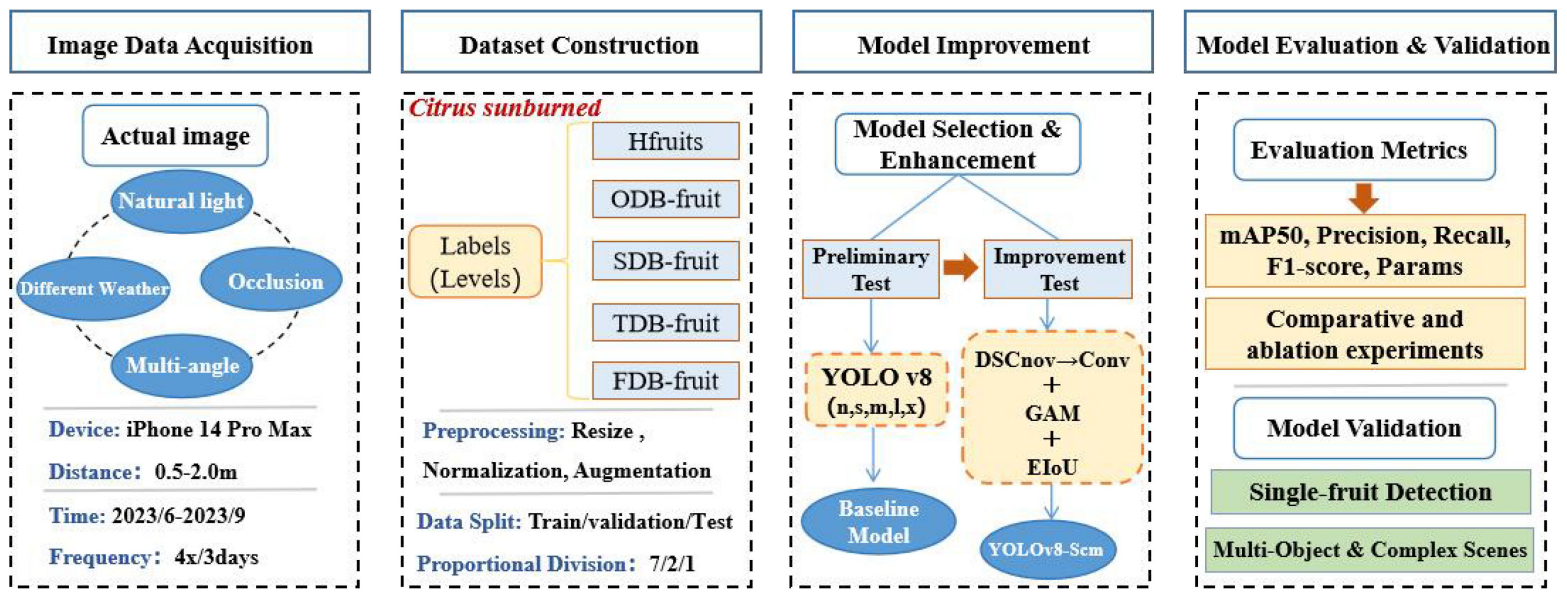
Structured workflow for citrus sunburn classification based on improved YOLOv8n.

The overall workflow of this study consists of four main stages: image data acquisition under natural orchard conditions, dataset construction with sunburn level labeling and preprocessing, model development and optimization based on YOLOv8n incorporating DSConv, GAM, and EIoU modules, and finally, model evaluation and validation using standard metrics such as mAP50, Precision, and Recall (**Figure 1**). This structured process ensures both effectiveness and applicability of the proposed method in real-world citrus sunburn detection scenarios. Each step is detailed in Sections 2.1 through 2.5.

**Figure 2:**
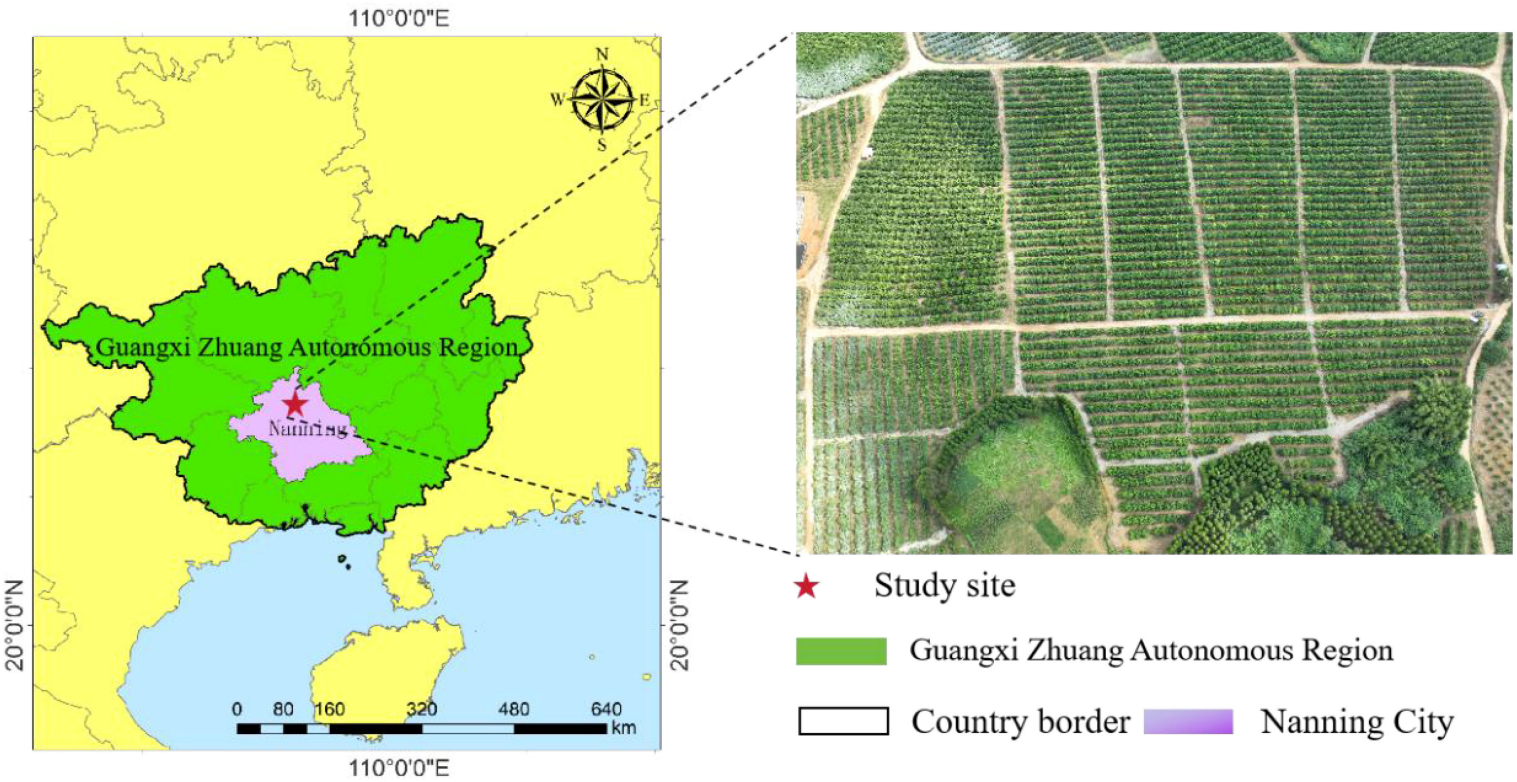
Geographical diagram of the citrus fruit collection areas.

This study was conducted in an Orah mandarin orchard located in Wuming District, Nanning City, Guangxi Zhuang Autonomous Region, China (*22°59′58″ N, 108°37′22″ E*). The orchard is located in the subtropical monsoon region (**Figure 2**). It covers an area of approximately 50 hectares with a row spacing of 4.5 m and plant spacing of 3.0 m.

**Figure 3:**
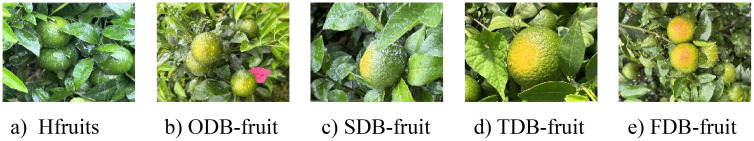
Sample images of citrus sunburn at different degrees. The criteria for this 5-level division are as follows: **(a)** Hfruits: The fruit is pure green without any color difference, categorized as normal and healthy. **(b)** ODB-fruit: The fruit has a slight yellowing, or a small part of it turns yellow, which indicates that the fruit was already green and had a trend of discolouration due to burns. **(c)** SDB-fruit: The fruit has obvious discolouration, but the proportion of the discolored area is relatively small, usually less than 25% of the light-facing area. **(d)** TDB-fruit: The fruit has a relatively large area of burns, but dry scars and scabs have not yet formed, accounting for 25%-66.7% of the area. **(e)** FDB-fruit: The fruit has severe burns, and dry scars and scabs have formed, or the area of abnormal discolouration caused by the burns exceeds 66.7%.

Regarding citrus sunburn, although researchers from various countries have extensively studied it, few specific standards for grading citrus sunburn have been formulated. In this study, based on the actual experimental collection data, following Wang et al (Wang et al., 2020). and Hu et al (Hu., 2020), we divided the citrus sunburn damage grades into five categories: Hfruits (Healthy fruit), ODB-fruit (sunburning level 1 fruit), SDB-fruit (sunburning level 2 fruit), TDB-fruit (sunburning level 3 fruit), and FDB-fruit (sunburning level 4 fruit). **Figure 3** shows the samples of sun-burning fruit for each level.

### 2.1 Image acquisition

The data were gathered during the fruit expansion stage from June to September 2023, when the fruit was green. At this stage, the citrus fruits had not yet ripened or undergone color change, ensuring that natural ripening processes did not interfere with the assessment of sunburn. After sunburn, the fruit showed discolouration, turning yellow and brown, which was in clear contrast to the green fruit.

The data collection was conducted every three days at three fixed time intervals: 07:00–09:00, 12:00–14:00, and 16:00–18:00, under different lighting conditions. The acquisition device was an iPhone 14 Pro Max mobile phone with a pixel resolution of 4536×8064 and a shooting distance of 0.5-2.0m. The pictures were saved in JPG and PNG formats. To ensure that the experimental data were extensive and representative, the data were collected from Orah mandarin orchards in a natural environment. This environment included various complex conditions such as direct sunlight, shade obstruction, and rainy and sunny days.

### 2.2 Dataset creation

To create a dataset to facilitate the processing and analysis of collected data in subsequent experiments, 2,099 pictures of citrus sunburn fruits at different levels were used. First, the original images were uniformly cropped to two sizes, 1280×1280 and 640×640 pixels. Then, LabelImg software was utilized for labeling. According to the different levels of citrus sunburn, a total of five labels (Hfruits, ODB-fruit, SDB-fruit, TDB-fruit, FDB-fruit) were set, with a total of 5390 boxes. The dataset was partitioned into three subsets: a training set, a validation set, and a test set, in a ratio of 7:2:1. A summary of the dataset properties, annotation methods, and preprocessing steps is presented in **Table 1**.The numbers of citrus fruits with different levels of damage in each dataset are presented in **Table 2**.

**Table 1:** Dataset details and preprocessing pipeline for citrus sunburn detection.

Dataset Property	Description
Total images	2099
Number of labels	5390
Sunburn classes	5(levels 0-4)
Annotation tool	Labelimg
Preprocessing	Resize to 640×640, 1280×1280 normalization, augmentation (flip, rotate, brightness adjust)
Data split ratio	Train: 70%, Val: 20%, Test: 10%

**Table 2:** Detailed composition of the citrus fruit sunburn identification, division and detection datasets.

Sample name	Training set/box	Validation set/box	Test set/box	Total/box
Hfruits	1325	245	83	1653
ODB-fruit	852	244	121	1217
SDB-fruit	751	215	108	1074
TDB-fruit	446	128	64	638
FDB-fruit	566	161	81	808
Total	3940	993	457	5390

The dataset used in this study will be publicly released upon the acceptance and publication of this article. It will be made available for academic research and educational purposes only. In addition, we plan to continuously expand the dataset by including more citrus varieties, sunburn levels, and images captured under different environmental and lighting conditions. Future versions of the dataset will be uploaded to the same repository to support ongoing research in fruit stress identification and precision agriculture.

### 2.3 YOLO v8 model structure and preliminary test

#### 2.3.1 YOLO v8 model structure

YOLO v8 is one of the most widely used object detection models (Jocher et al., 2023). It consists of three parts: the backbone network, the head network, and the neck network. Compared with earlier versions, YOLOv8 has several advantages. It has a better CSPNet model architecture and an improved loss function CIoU. Moreover, it supports detection, segmentation, and classification tasks. For different goals and usage scenarios, YOLOv8 is divided into five different versions: YOLOv8n, s, m, l, and x.

This study mainly focuses on citrus orchards in complex natural environments (Xu et al., 2023). To meet the need for accurately and rapidly detecting citrus sunburn in orchards, YOLOv8n, which has the least amount of parameter calculation, was selected as the baseline model for subsequent improvement and optimization, considering the subsequent lightweight deployment of the model.

#### 2.3.2 Preliminary test

In this study, We conducted a pre-test to evaluate the performance of five versions of YOLOv8 on the established dataset. This was done to verify the rationality and effectiveness of the selected model. The experimental results are presented in **Table 3**. It also presents the overall model mAP50, precision rate (P), and recall rate (R). Among these evaluation indicators, YOLOv8m performs the best, with mAP50, P, and R scores of 93.2%, 90.3% and 87.6%, respectively. In this study, the selected YOLOv8n model is lightly inferior to the other versions of the model in terms of the evaluation indicators of mAP50, Precision (P), and Recall (R). However, it can fully meet the monitoring requirements in the experiment and application. Moreover, the number of model parameters of YOLOv8n is 3.01M, far lower than those of other models. This gives it a great advantage in achieving weight reduction improvement. The objective of this study is not merely to pursue optimal detection precision but, more importantly, to ensure the model’s lightweight design and enhanced real-time performance within a reasonable precision range. Therefore, this study selects YOLOv8n as the baseline model for subsequent improvement and optimization.

**Table 3:** Pre-experiment results of the baseline model of different versions of YOLOv8.

Models	Single-label accuracy					mAP50/%	P/%	R/%	Params/M
	Hfruits	ODB-fruit	SDB-fruit	TDB-fruit	FDB-fruit				
YOLO v8n	87.1	78.6	85.3	88.4	85.8	90.7	85.1	87.1	**3.01**
YOLO v8s	**93.2**	85.6	88.0	**91.2**	90.5	91.9	89.7	85.3	11.12
YOLO v8m	92.4	**86.5**	86.7	90.1	**95.7**	**93.2**	**90.3**	**87.6**	26.58
YOLO v8l	92.1	84.5	86.7	86.3	89.0	92.2	87.7	87.2	44.92
YOLO v8x	92.3	85.0	**88.1**	86.4	91.4	91.9	88.6	86.1	70.18

Bolded values represent the highest performance among all models for the corresponding evaluation metric.

### 2.4 Model improvement

**Figure 4:**
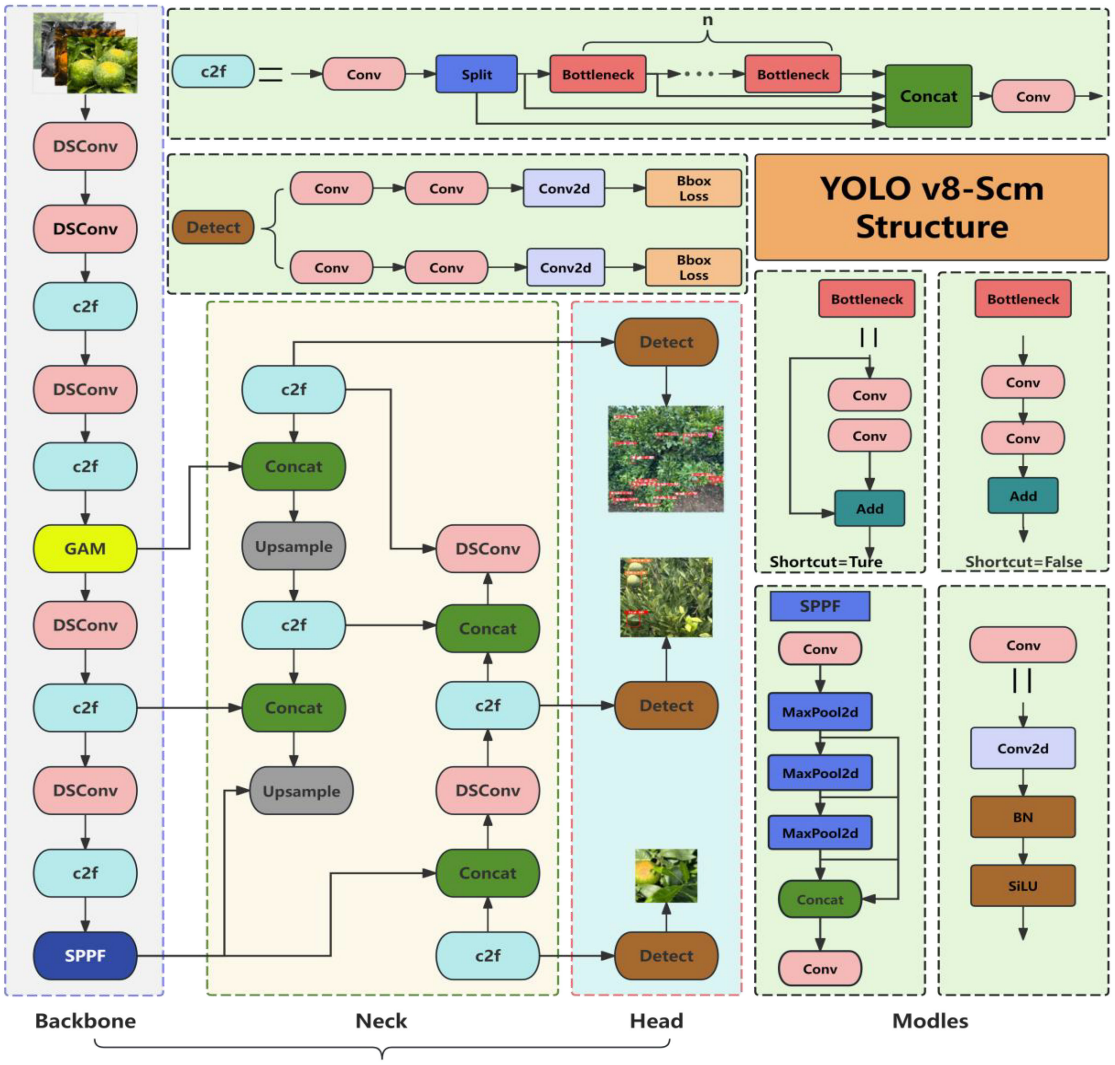
Frame structure of the YOLOv8-Scm model.

The YOLOv8n baseline model performs well in terms of its lightweight design and real-time capabilities. However, in natural and complex scenes such as those with strong light and chaotic backgrounds, its recognition precision and effective detection cannot be guaranteed, and missed detection and false detection may occur (Zhang et al., 2024b). To endow the model with lightweight and accurate real-time monitoring performance and enhance its ability to identify and classify citrus sunburn fruits in complex environments, an improved model based on YOLOv8n, named YOLOv8-Scm (YOU ONLY LOOK ONCE v8-Sunburn citrus monitoring), is proposed. We selected DSConv, GAM, and EIoU after evaluating several candidate modules for balancing model precision and computational efficiency.The details are as follows. As shown in **Figure 3**, according to the application requirements of the scenario, three improvements are made to the original YOLOv8n model. First, We replaced the Conv module with the DSConv module (Nascimento et al., 2019). This replacement aims to achieve lightweight and efficient calculations of the model and reduce the consumption of computing resources. Second, the global attention mechanism (GAM) (Liu et al., 2021) is introduced. It helps improve the focus on global context information, filter complex background interference, and improve the detection precision and robustness of the model for sunburning fruits of various sizes. Finally, to reduce the problem of large detection errors caused by the inconsistent sizes and diverse distributions of citrus fruits in the natural environment, the EIoU bounding box regression loss function (Zhang et al., 2022) is introduced. This function improves the ability of surface feature extraction and identifying citrus fruits in challenging environments. The model can more precisely adjust the object box, accelerate model convergence, and reduce the occurrence of missed detection and false detection, thereby improving the model’s overall performance. The YOLOv8-Scm network structure is based on the above improvements, as shown in **Figure 4**.

#### 2.4.1 Distribution-shifted convolution

**Figure 5:**
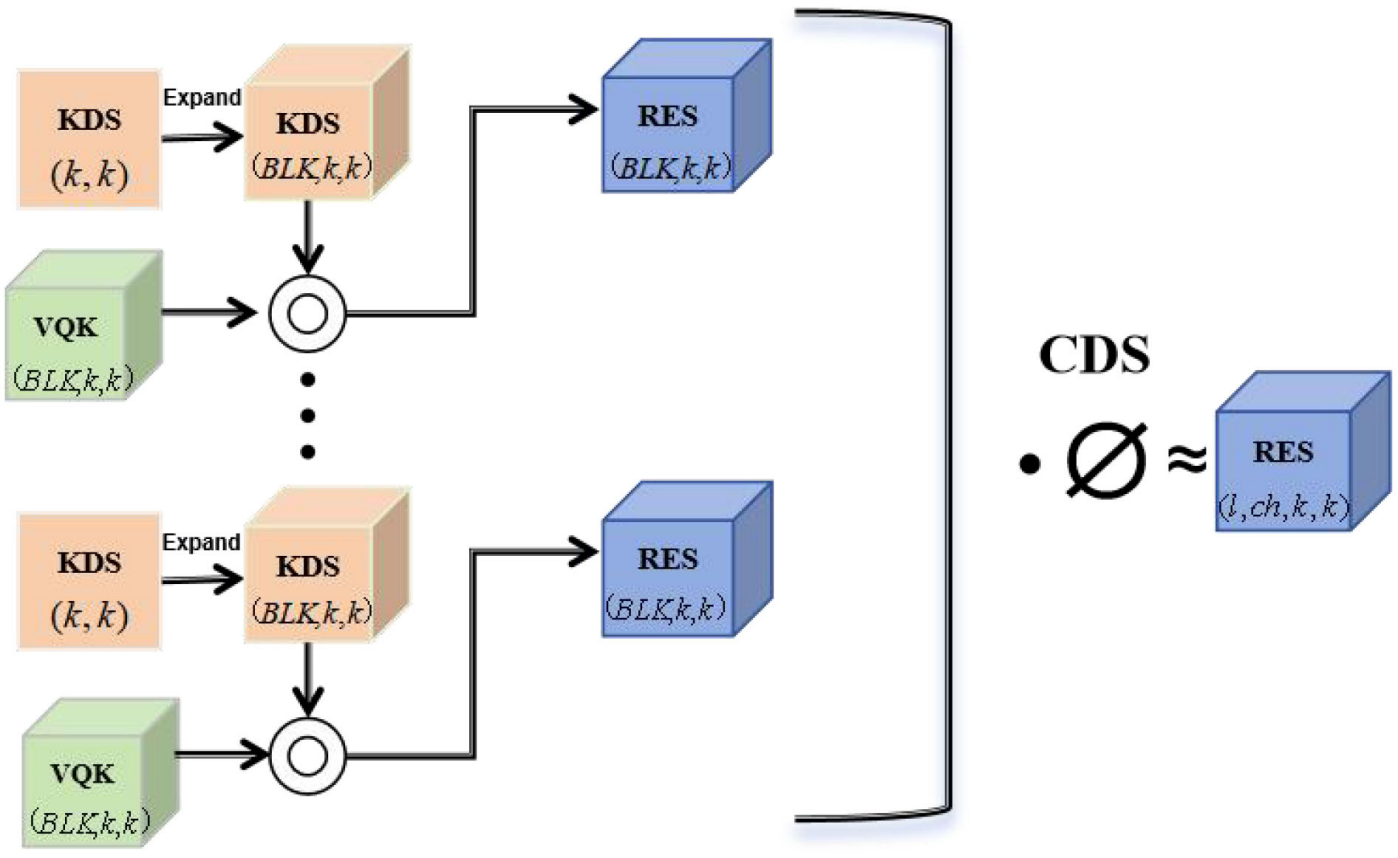
DSConv module structure.

The DSConv, an efficient variant of the convolutional layer proposed by Nascimento et al (Nascimento et al., 2019), can lead to lower memory consumption overhead and higher computation speed. To ensure the light weight and high-speed operation performance of the model, this paper selects DSConv to replace the Conv module as the backbone feature extraction network of YOLOv8n. The structure of the module is illustrated in **Figure 5**.

DSConv consists of two main components: a variable quantized kernel (VQK) and a kernel distribution shift (KDS). In VQK, only the quantized integer values are stored. By applying the distribution offset mechanism based on kernels and channels, to retain the output characteristics as closely as possible to those of the original convolution operation, are retained as much as possible. The distribution shifter is implemented by two tensors: a KDS and a channel distribution shifter (CDS). The structural characteristic of DSConv lies in its capacity to effectively optimize the quantized feature distribution. That is, it can reduce computational consumption as much as possible while ensuring the precision of the model. This enables it to adapt to the complex lighting conditions and the appearance characteristics of fruit in the natural environment, thereby obtaining more accurate models. In this way, a network model with higher speed, greater robustness, and better real-time performance can be achieved.

#### 2.4.2 Global attention mechanism

Under natural conditions, citrus plants have a low degree of differentiation from their surroundings. Some citrus fruits are obscured by branches and leaves, making them difficult to identify and locate. When monitoring small targets and objects with high feature similarity in natural and complex scenes, the traditional YOLOv8n model performs unsatisfactorily. Woo et al (Woo et al., 2018). proposed the convolutional block attention module (CBAM)to improve the performance of Convolutional Neural Networks (CNNs). CBAM combines channel attention and spatial attention mechanisms and can concentrate on and acquire features, for example, the shape, color, and texture of citrus fruits. However, although the attention mechanism can improve the model’s monitoring performance, an increased number of parameters leads to greater computational overhead, which affects the performance of the model. Liu et al (Liu et al., 2021). proposed an improved GAM built upon the CBAM. GAM captures the global information of the channel, width, and height through 3D feature arrangement to enhance the ability of cross-dimensional information expression. It also optimizes computation efficiency through group convolution of channel shuffling. A lightweight design is used to reduce the increase in the additional parameters introduced by the spatial attention module, significantly improving the model’s performance in relation of weight reduction and computational efficiency. For this reason, this study introduced GAM into the YOLOv8n model to globally optimize the performance and precision of citrus identification in complex environments.

The GAM consists of two main parts: the channel attention submodule and the spatial attention submodule. It continues to use the sequential processing method of CBAM. The difference is that it introduces the modelling of global context information by globally performing on the entire feature map. Pooling is used to capture the global information of the entire image. The workflow and formula steps are as follows:

##### 2.4.2.1 Channel attention module

Global pooling: Global average pooling is applied to each channel. The detailed process formula is as shown in Equation 1:


(1) $$F_{\text{avg}}=\frac{1}{H\times W}\sum_{\text{i}=1}^{H}\sum_{\text{j}=1}^{W}F_{1}(i,j)$$

where $F_{\text{avg}}$ represents the pooled feature vector, $F_{1}\in R^{C\times H\times W}$ stands for the input feature map, c is the channel index (i.e., the channel count of the input feature map), H is the feature map height, W is the width of the channels, and i and j are the row index and column index of the feature map, respectively.

Multi-layer perceptron (MLP): The pooled channel description is sent through a two-layer MLP, after which the weight of each channel is output. The process formula is as shown in Equation 2:


(2) $$M_{C}(F_{1})=\sigma (W_{2}\cdot R\text{eLU}(W_{1}\cdot F_{\text{avg}}))$$

**Figure 6:**
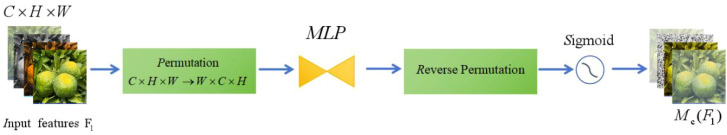
GAM-Channel attention submodule.

Where $W_{1}$ and $W_{2}$ represent the MLP weights, and where σ represents the sigmoid activation function which compresses the output value to the range between 0 and 1 Calculate and take $M_{C}(F_{1})$ along with the input features $F_{1}$ . Then perform channel-by-channel multiplication to generate the enhanced feature maps. The workflow diagram of channel attention is as follows: **Figure 6**:

##### 2.4.2.2 Spatial attention module

1. Pooling operation: First, global average pooling and max pooling are applied to the input features. Subsequently, the two pooling results are spliced along the channel dimension to generate a fused feature representation and a spatial description;
2.
7×7 Convolution operation: The spliced pooled results are passed through 7×7 convolutional layers to generate spatial attention maps Equation 3:

(3) $$M\text{s}(F2)=\sigma (f^{7\times 7}([AvgPool(F_{2});MaxPool(F_{2})]))$$

where $f^{7\times 7}$ the convolution operation of 7×7 , []; represents channel splicing and where is the sigmoid activation function.

**Figure 7:**
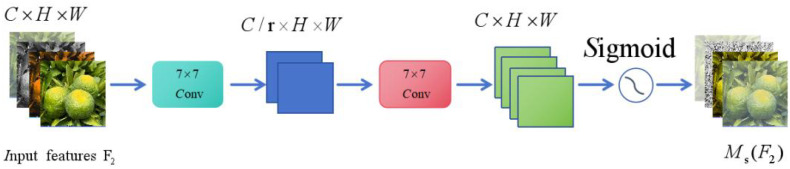
GAM-Spatial attention submodule.

**Figure 8:**
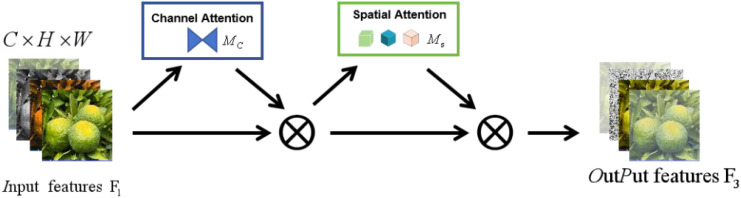
Overview of the GAM.

The spatial attention $M_{S}(F_{2})$ is generated, and the characteristics $F_{2}\in R^{C\times H\times W}$ are also formed. The final output features are obtained by multiplying each element. The workflow diagram of the spatial attention module is as follows: **Figure 7**:

In summary, the process and structure diagram of the GAM are as follows: **Figure 7** and Equation 4 show the following:


(4) $$F_{2}=M_{C}(F_{1})⊗F_{1}$$

where $M_{C}(F_{1})$ is the channel attention map, ⊗ represents the operation of multiplying the element-by-channel, and $F_{2}\in R^{C\times H\times W}$ is a feature of the channel after enhancement.


(5) $$F_{3}=M_{S}(F_{2})⊗F_{2}$$

where $F_{2}\in R^{C\times H\times W}$ is the channel-enhanced feature, $M_{S}(F_{2})$ is the spatial attention map, ⊗ represents element-by-channel multiplication, and the final output features can be obtained after the above steps $F_{3}$ See Equation 5 and **Figure 8** for details.

#### 2.4.3 Enhanced Intersection over Union loss function

In the recognition detection algorithm, the loss function measures the model’s bounding box positioning accuracy, prediction confidence, and object classification and identification precision. By calculating the difference between the predicted value and the actual value (i.e., the weighted sum), it guides the optimization of model parameters. This helps to minimize the error, improve the model’s generalization ability, and prevent over fitting during the training process.

The selection and design of the loss function directly affect the model’s performance. Regarding this, the original CIoU (Complete Intersection over Union) loss function (Du et al., 2021) is improved based on the EIoU loss function (Zhang et al., 2022). This improvement is mainly reflected in the following aspects: refined constraint on the difference in the length and width of the bounding box, enhanced convergence speed and detection precision, and enhanced optimization for small object ability and more balanced loss distribution. The specific formula is as shown in Equation 6:


(6) $$\begin{array}{l} L_{EIOU}=L_{IOU}+L_{dis}+L_{asp}\\ =1-IOU+\frac{\rho^{2}(b,b^{gt})}{{\left(w^{c}\right)}^{2}+{\left(h^{c}\right)}^{2}}+\frac{\rho^{2}(w,w^{gt})}{{\left(w^{c}\right)}^{2}}+\frac{\rho^{2}(h,h^{gt})}{{\left(h^{c}\right)}^{2}} \end{array}$$

where w,h represent the width and height of the prediction box, respectively; $w^{gt},h^{gt}$ represent the width and height of the target box, respectively; $w^{c},h^{c}$ represent the width and height of the minimum bounding box containing the prediction box and the target box, respectively; and $\rho^{2}(b,b^{gt})$ represents the squared difference between two values, usually the square of the Euclidean distance.

## 3 Results and analysis

### 3.1 Experimental platform and training parameter settings

In this study, the following experimental equipment was used. The operating system was Ubuntu20.04. The processor was an Intel Xeon(R) Silver4314 CPU@2.40 Hz×64, and the graphics card was NVIDIA A10 24G. The compilation language was Python 3.8.5, with Pycharm. 2023 as the compilation software. The deep learning framework was torch-2.0.0, and CUDA was utilized to accelerate the training.

During the experiments, for each iterative training of the model, the number of samples was set to 16. The number of iterative training rounds (epochs) was set to 300, and the Adam optimizer was used as the optimizer. The experimental results are presented in **Table 4**.

**Table 4:** Hyperparameter configuration of the deep learning experiment.

Training parameters	Details
Epochs	300
Batch_Size	16
Input	640*640
OPtimizer_Type	Adam
Momentum	0.937
Weight decay	0.0005

#### 3.1.1 Algorithm performance evaluation

In this study, for the achievement of classification and ranking of citrus fruits with varying degrees of sunburn under complex natural conditions, it is necessary to identify and classify the citrus fruits with different levels of sunburn and perform quantitative calculations. To assess the performance of the YOLOv8-Scm model in identifying citrus sunburn fruit damage levels, three quantitative indicators were used as performance indicators: mAP50, Precision (P), Recall (R), as reference bases for the evaluation. Additionally, Params (model parameters) was used to evaluate the parameter complexity of the model to measure the model’s weight reduction.

The “Precision (P) is used to evaluate the prediction precision of the model, which is the ratio of the samples predicted by the model to be in the positive category to the samples actually in the positive category. The calculation formula is as shown in Equation 7:


(7) $$P=\frac{T_{p}}{T_{p}+F_{p}}\times 100\%$$

The Recall (R) measures the proportion of all actual positive samples correctly predicted by the model. The formula for its calculation is as shown in Equation 8:


(8) $$R=\frac{TP}{T_{p}+F_{n}}\times 100\%$$

The F1-score is the harmonic mean of precision and recall, reflecting the overall performance of a model by balancing its accuracy and completeness in classification tasks; a higher value indicates better detection capability.The formula for its calculation is as shown in Equation 9:


(9) $$F_{1}=2\times \frac{P\times R}{P+R}$$

The mAP50 refers to calculating the average precision (AP) for each category when the IoU threshold is 0.5, and then averaging all the categories. (IoU = 0.5 means that the detection is considered correct when the overlap area between the predicted bounding box and the ground-truth bounding box accounts for at least half of the area of their union, i.e., A∩B>0.5). A larger value represents a higher average precision of the model, that is, better detection performance. The calculation process is shown in Equations 10–12.


(10) $$IOU=\frac{A\cap B}{A\cup B}$$

(11) $$AP=\int_{0}^{1}P\cdot RdR$$

(12) $$\text{m}AP@50=\frac{1}{N}\sum_{i=1}^{N}AP_{i}$$

A∩B denotes the overlapping area between the predicted and ground-truth bounding boxes, while A∪B stands for the union area of these two.

Tp (True positives) refers to true examples, specifically, the count of samples where the model correctly predicts as the positive category.

Fp (False-Positives) are false-positive examples, meaning the amount of samples in which the model wrongly predicts the negative category as the positive category.

FN (False-Negatives) refer to instances where the model incorrectly classifies a sample that actually belongs to the positive category as negative.

### 3.2 Training results of the YOLOv8-Scm model

**Figure 9:**
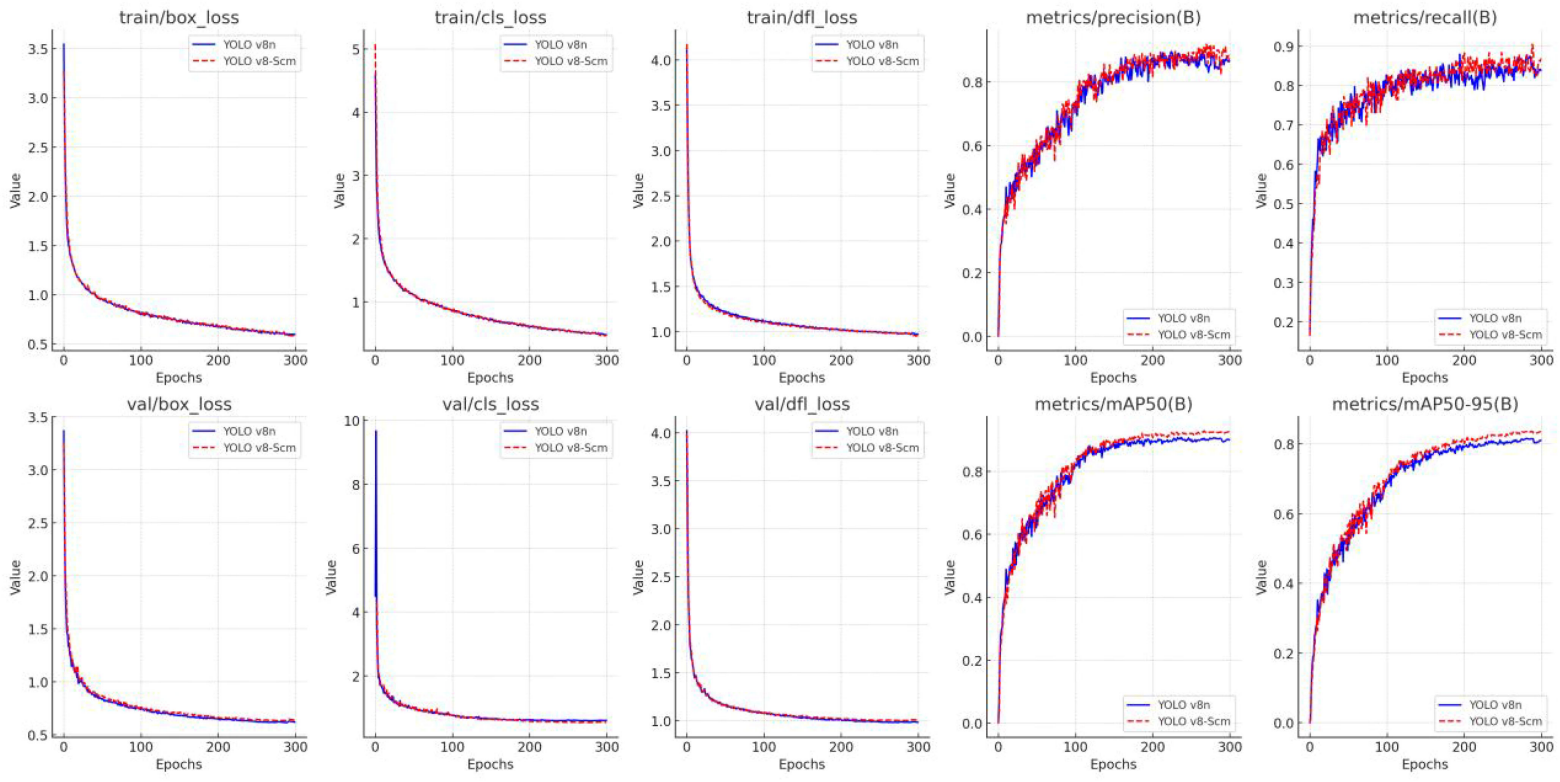
Comparison of the results of the YOLO v8n model before and after improvement.

As illustrated in **Figure 9**, the improved YOLOv8-Scm model outperforms the initial YOLOv8n model in all indicators, demonstrating higher detection performance and robustness. Firstly, regarding the model convergence speed, YOLOv8-Scm is slightly faster than YOLOv8n and has a lower loss value in the middle and late stages. This implies that the improved model is more accurate in the predicting of object bounding boxes and it has better object classification ability and model generalizability. Secondly, in terms of P, the average value of YOLOv8-Scm is 75.25%, with a maximum value of 91.92%. In contrast, the average value and maximum P of YOLOv8n are 74.43% and 89.62%, respectively. This shows that YOLOv8-Scm has a stronger ability to avoid false detection and can produce more accurate identification results. Thirdly, regarding the recall rate, the average value of YOLOv8-Scm is 79.05%, with a maximum of 90.50%, both of which are higher than those of YOLOv8n (78.52% and 87.96%, respectively). This reflects that YOLOv8-Scm has a more accurate Recall rate and better target identification ability. In addition, the average mAP50 value is 81.16% with a maximum value of 92.91%, while YOLOv8n achieves an average mAP50 of 80.04% and a maximum of 90.81%. For the more stringent mAP50–95 indicator, the average value of YOLOv8-Scm is 69.96% and the maximum value is 83.75%, which are higher than those of the original YOLOv8n model (with an average value of 68.62% and a maximum value of 81.59% respectively).

In summary, YOLOv8-Scm has been comprehensively improved in terms of P, R and generalization ability, especially for the key indicator mAP50. This result demonstrates the effectiveness of the model improvements proposed in this study. The YOLOv8-Scm model, which can complete multi-target detection tasks in complex natural environments, has even greater application potential and advantages.

### 3.3 Ablation experiment

In this study, the aim is to develop a lightweight detection algorithm for identifying sunburned citrus fruits in orchards by improving the original YOLO v8n model. The primary improvements are as follows: Firstly, replacing the Backbone feature extraction and compression input Conv module with the DSConv module. Second, the EIoU loss function is used to replace the CIoU loss function. Third, introduce the GAM global attention mechanism. To comprehensively evaluate the model’s performance, four groups of ablation experiments were carried out. These include the original model without any module improvements, the model with a single module added, the model with two modules added, and finally, the model with all improvements incorporated.

All ablation experiments were performed under identical training conditions, using the same dataset and training parameters. This was done to investigate the impact of adding different modules on the performance of the baseline mode and effectively clarify the effectiveness of each module improvement in the model.

**Table 5** shows that adding different modules and their combinations can improve all the evaluation indicators of the model. When using the combination of DSConv+EIoU+GAM, the model’s performance reaches the optimal balance. The mAP50 increases to 92.7%, which is 2.0% higher than the base model’s 90.7%. Moreover, the P reaches 86.6% and the R reaches 87.2%, which are significantly better than the base model’s 85.1% and 87.1% respectively. This indicates that the model’s classification ability and bounding box positioning ability for citrus sunburn fruit have been significantly improved. In addition, the amount of parameters of the improved model only slightly increases by 0.182M from the original model’s 3.01 M. Although the computational complexity is increased slightly, the overall performance is significantly improved, which ensures the model’s lightness.

**Table 5:** Ablation experiments based on the YOLO v8n model.

DSConv	EIoU	GAM	Single-label accuracy					mAP50%	P/%	R/%	Params/M
			Hfruits	ODB-fruit	SDB-fruit	TDB-fruit	FDB-fruit				
×	×	×	87.1	78.6	85.3	**88.4**	85.8	90.7	85.1	87.1	3.010
**√**	×	×	89.2	**81.9**	82.3	86.5	86.3	91.1	85.2	86.2	**3.007**
**√**	**√**	×	90.3	**81.9**	**88.3**	87.6	85.7	91.4	**86.8**	84.4	**3.007**
**√**	**√**	**√**	**91.9**	80.7	85.0	87.3	**88.2**	**92.7**	86.6	**87.2**	3.192

Bolded values represent the highest performance among all models for the corresponding evaluation metric.

Based on the above results, the synergy of module combinations effectively improves the precision of classifying and identifying citrus sunburn fruits in complex natural scenes, enhances the model’s light weight and robustness. This advantage is especially well-suited for deployment on resource-limited hardware devices and mobile phones in actual field applications.

### 3.4 Comparative test

To better demonstrate the model’s overall performance through comparison, the proposed YOLOv8-Scm model was compared with mainstream models for state-of-the-art (SOTA) results, thus proving the superiority of its performance. The mainstream lightweight object detection models for comparison are SSD (Liu et al., 2016), FasterR-CNN (Ren et al., 2016), YOLOv5n (Wu et al., 2021), YOLOv7-tiny (Cheng et al., 2023), YOLOv8n, and YOLOv10n (Wang et al., 2024). Throughout the training phase, the default input image resolution was used, the training parameters were kept the same, and the same hardware environment and dataset were employed for training. This ensured fairness by minimizing interference of other non-human factors and controlling variables to make the training results more informative. For specific results, please refer to the table below.

To comprehensively evaluate the performance and computational efficiency of the proposed YOLOv8-Scm model, **Table 6** presents a comparison with several mainstream object detection algorithms commonly used in fruit detection tasks. Traditional models such as SSD and Faster R-CNN achieve reasonable accuracy but are computationally intensive and unsuitable for real-time deployment. Lightweight models like YOLOv5n and YOLOv7-tiny offer faster inference but are generally limited to binary or low-level classification and perform less reliably under complex conditions such as occlusion and variable lighting.

**Table 6:** Performance comparison experiment of mainstream object detection models.

Models	mAP50/%	P/%	R/%	Params/M	F1-score	FPS
SSD	86.7	79.5	83.0	24.2	81.21	93.299
Faster R-CNN	89.8	73.2	89.4	136.8	80.48	22.962
YOLO v5n	86.2	82.8	88.9	**1.8**	85.74	128.205
YOLO v7-tiny	87.3	82.1	**89.5**	6.0	85.65	86.207
YOLO v8n	90.7	85.1	87.1	3.0	86.10	**135.14**
YOLO v10n	90.9	84.7	85.7	26.96	85.20	89.286
Ours	**92.7**	**86.6**	87.2	3.2	**86.90**	117.647

Bolded values represent the highest performance among all models for the corresponding evaluation metric.

Compared with these models, YOLOv8-Scm supports fine-grained five-level classification and demonstrates superior robustness and stability in real orchard environments. Importantly, it achieves an mAP50 of 92.7% with only 3.2M parameters, and reaches a real-time inference speed of 117.647 FPS on a standard GPU. These results highlight a favorable trade-off between detection accuracy and computational demand, making the model more applicable to real-time, mobile, or embedded deployment scenarios for precision agricultural stress monitoring.

The task of identifying and detecting citrus sunburn fruit in natural scenes is complex, as it involves situations like fruit of different sizes and occlusions. By comprehensively comparing the indicators in the above table, we find that the YOLOv8-Scm model we propose has an edge in this task. Specifically, in terms of the mAP50, P, and R indicators, YOLOv8-Scm outperforms other mainstream detection models. When compared with the two traditional detection algorithms, SSD and Faster R CNN, the mAP50 of YOLOv8-Scm increases by 6.01% and 2.87% respectively; the P increases by 7.1% and 13.4%; the R increases by 4.2% and -2.2% respectively. Meanwhile, the amount of Params/M is reduced by 20.96 M and 133.58 M, respectively. This indicates that all the performance parameters of YOLOv8-Scm have been significantly improved and the number of model parameters has been significantly reduced, ensuring its lightweight advantage.

When compared with YOLO v5n, YOLO v7-tiny, and YOLO v8n, the mAP50 of YOLOv8-Scm is increased by 6.5%, 5.4%, and 2%, respectively, and the P is increased by 3.8%, 4.5%, and 1.5%, respectively. The R of YOLOv8-Scm is 87.2%, which is slightly lower than those of these four models (ranging from -0.1% to 2.3%), but this does not lead to a significant loss in model performance. When compared with the newly released YOLOv10n model, YOLOv8-Scm has significant advantages. Also, the number of parameters of the YOLOv8-Scm model is 23.77 M lower than that of the YOLOv10n model. In summary, the findings fully indicate the advantages of the YOLOv8-Scm model.

### 3.5 Model validation for visualization

**Figure 10:**
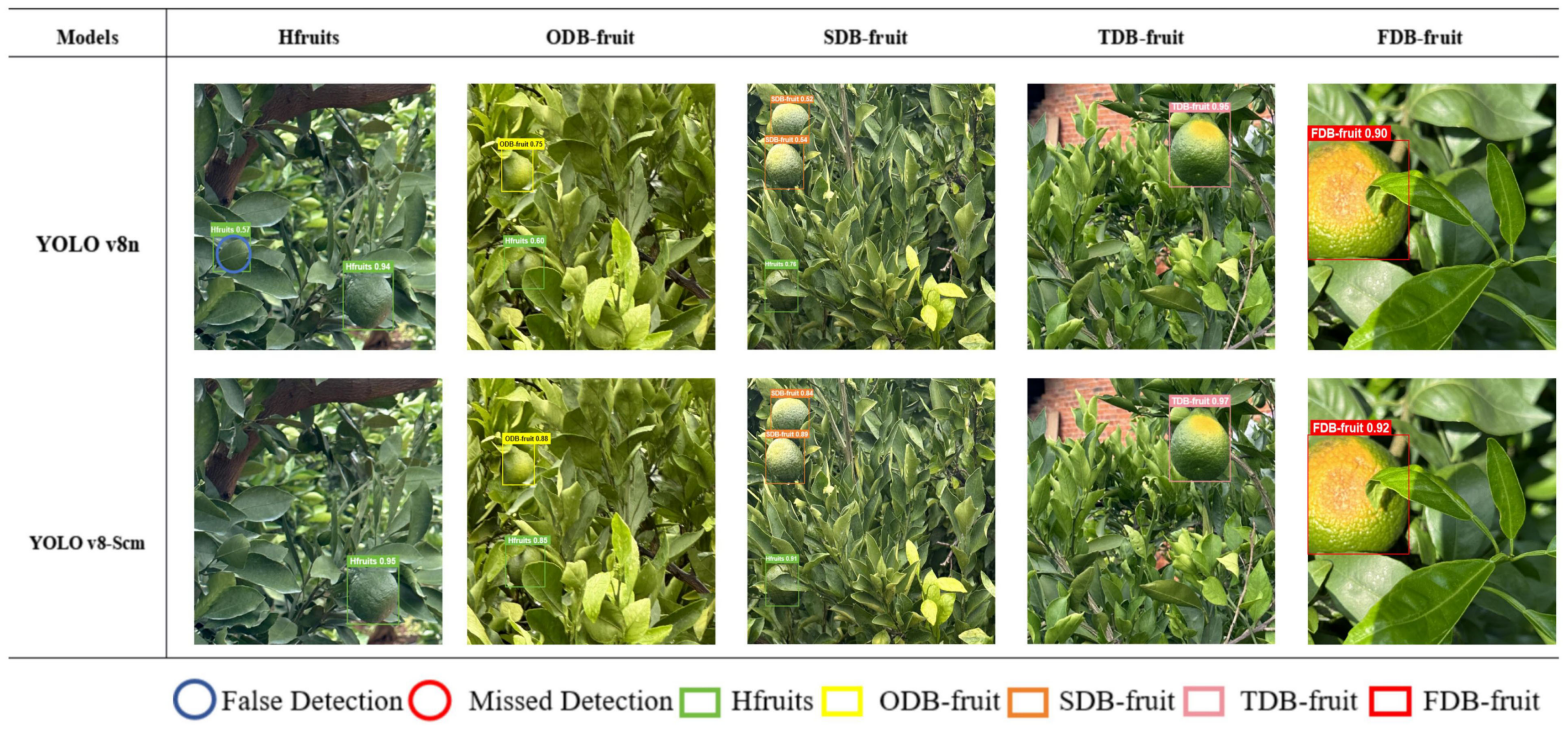
Test results for each grade of sunburn fruit.

In citrus orchards, under natural conditions, high-precision detection for the identification and classification of citrus sunburn fruits is crucial for real-time monitoring with panoramic cameras. To evaluate the performance of the improved YOLOv8n, 2 groups of experimental pictures of citrus sunburn fruits (five pictures in each group) were used for testing and evaluation. The first group of pictures was utilized to identify a small number of fruits, aiming to test the detection precision of different-level labels (**Figure 9**). The second group of pictures was used for the identification and grading of multiple fruits and sun-burned fruits under various complex environmental disturbances (**Figure 10**). This was to test the multi-object classification and identifcation capabilities of algorithms with different sizes and different labels, as well as the detection precision in complex environments. For the improved model, the precision rate (P) was 86.6%, the recall rate (R) was 87.2%, the mAP50 was 92.7%, and the model parameter size was 3.192 M.

Representative images of the identifcation labels for each grade of citrus sunburn fruit selected from the first group are shown in **Figure 10**.

The Figure above illustrates a visual comparison of the detection and recognition outcomes between the enhanced YOLOv8-Scm model and the original YOLOv8n model. The main detection method is for a single fruit or a small amount of fruit. Compared with the YOLO v8n model, the latter has problems such as false detection and low dentinfication precision. For example, it may identify a leaf as a green and healthy fruit. However, on the same set of picture data, the YOLOv8-Scm model performs better. There is no missed detection or false detection, and it has a high confidence level. This indicates that the YOLOv8-Scm model has a greater effect on citrus sunburn in the natural environment and has accurate identification ability when grading fruit.

**Figure 11:**
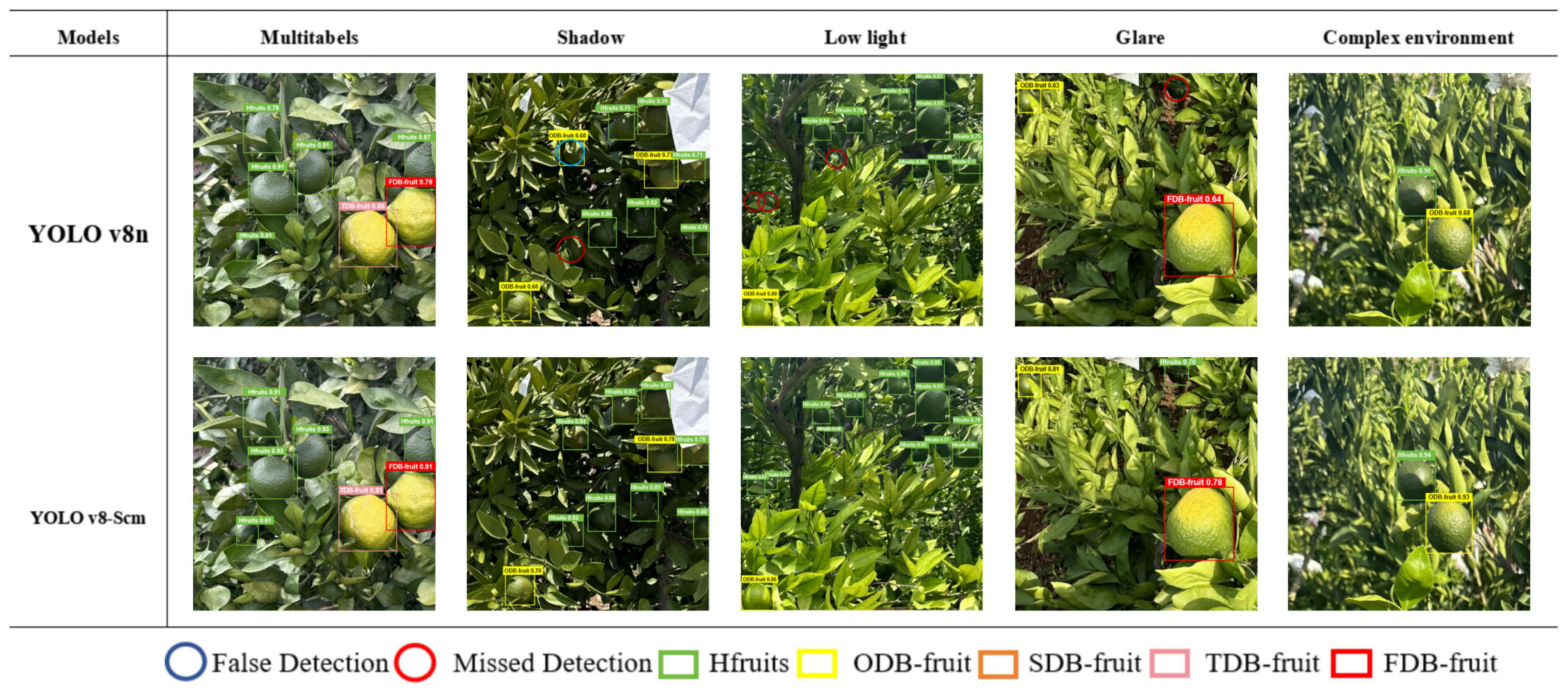
Detection results for complex environments and mixtures of diferent levels.

Citrus plants are mainly in the open air, so the conditions are complex. It is necessary to accurately monitor and identify multiple citrus fruits of different sizes and degrees of sunburn. There are problems like multilevel citrus sunburn interference identification, shading of branches and leaves, and light interference. After achieving accurate identification of single fruit labels, the second group of tests is carried out. Based on five conditions namely fruit mixing with multiple citrus sunburn levels, shadow and backlight, low light conditions, strong light irradiation, and branch and leaf obstruction, the second set of pictures is used for visualization comparison tests. Taking the fruit test results with multiple citrus sunburn levels in **Figure 11** as an example, citrus can be accurately identified and graded from healthy fruit to four levels of citrus sunburn regardless of the existence of obstruction, light interference, and fruits of different sizes. The results are effective, missed detection can be effectively avoided, and the identification and detection tasks can be completed quickly and accurately.

As shown in **Figure 11**, in a complex natural environment (with interference such as occlusion, strong light, branch and leaf obstruction), when identification is based on YOLO v8n, the models attention to citrus sunburn fruits and identification precision are slightly reduced, which leads to false detection and missed detection.

For instance, in Shaded environments, missed detections and erroneous detections occurs. When there is strong light or low-light interference, some small objects missed detection obscures the fruit. In comparison, the YOLOv8-Scm optimization model, which is obtained on the basis of the improved YOLOv8n, performed well in the above tests. There were no missed or false detections of citrus fruits, and the model had a high degree of confidence and high identification precision, as well as accurate grading ability. This result indicates that the optimized model is superior in identifying citrus sun-burning fruits and can efectively improve the monitoring ability and identification precision of citrus sun-burning fruits.

## 4 Discussion and conclusions

### 4.1 Discussion

Most existing monitoring and identification models for citrus trees mainly focus on two aspects: the identification of fruit number (Mirhaji et al., 2021; Cheng et al., 2023)and the identification of single ripe and immature fruits (Wang et al., 2024) (Lyu et al., 2022). Moreover, their identification labels are mostly divided into 1–3 classification labels. Currently, limited research has been conducted on identify- ing the disaster status of citrus sunburn fruits, and limited research on the identification of citrus sunburn damage. In addition, studies that combine machine vision technology and grading standards to classify citrus sunburn into five identification labels (0, 1, 2, 3, 4), have not been reported or conducted yet. When the same research object is subdivided into multiple classifications, many problems will occur, such as high similarity and difficulty in feature extraction. In this experiment, the improved YOLOv8-Scm model based on the YOLO v8n model can achieve identification and classification. It can accurately identify multiple grades of citrus sunburn fruits in natural scenes, and has high precision, strong robustness, and the ability to simultaneously target large and small objects, which are the characteristics of the monitoring. At the same time, this improved model can be deployed to the orchard panoramic camera to realize real-time monitoring and intelligent management of large orchard areas.

**Figure 12:**
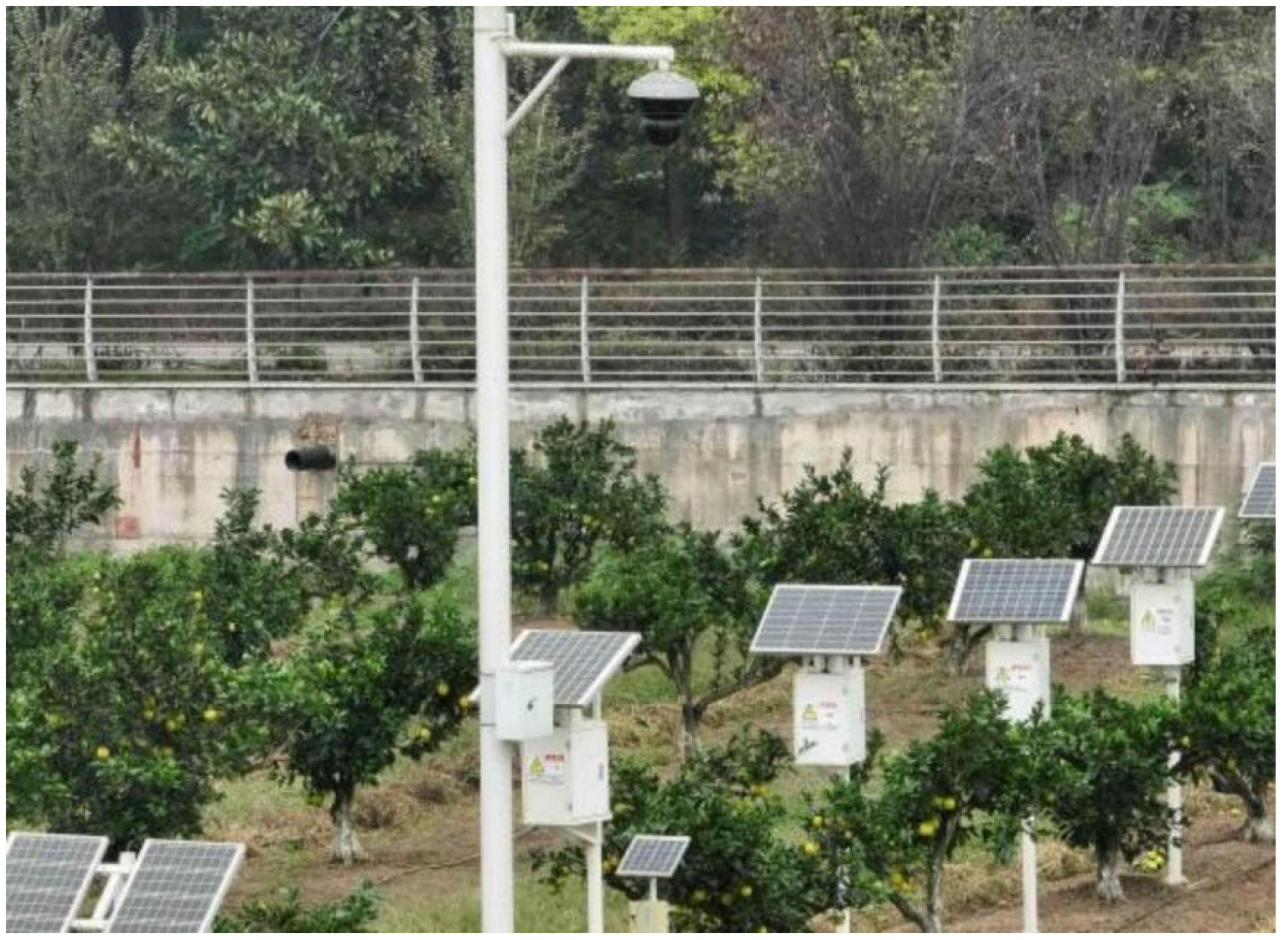
Deployment of a citrus sunburn panoramic camera based on the YOLOv8-Scm model in an orchard.

In the experiments, grading and labeling were susceptible to subjective judgment. The main reason was that the appearance features of the labels are similar, which afected the precision of feature extraction at different levels during model training to a certain extent. Regarding the citrus sunburn, it mainly occurs between June and September. During this period, when collecting and monitoring citrus sunburn, the citrus is in the fruit expansion stage and the target is easier to identify. At this time, the fruit is immature and green appearance, so it is easy to observe the citrus fruit that has been discolored due to sunburn. Moreover, the observations and data collection are not afected by the discolouration period in late October, thus avoiding the interference of certain natural factors. In follow-up research,deploying the model to more vectors is considered to achieve multivariate and multidimensional monitoring.The potential application scenarios for the future installation and deployment of this model in orchard production are illustrated in **Figure 12**.

Beyond citrus, the modular design of YOLOv8-Scm offers scalability for other plant stress detection tasks, such as apple and grape diseases. Similar challenges of fine-grained feature extraction and classification under complex backgrounds are prevalent in various agricultural applications.In addition, studies in other fields have explored related solutions to fine-grained classification problems. For instance, Chen et al. proposed a feature reconstruction method based on CBCT imaging combined with machine learning for cleft lip and palate classification, effectively addressing multi-scale structural variations and enhancing recognition accuracy (Chen et al., 2025). Although applied in medical imaging, their approach provides valuable insights into improving feature extraction and classification precision under challenging conditions. This aligns with the strategies adopted in YOLOv8-Scm, further supporting the effectiveness of attention mechanisms and optimized loss functions in complex recognition tasks.

Although the proposed YOLOv8-Scm model effectively identifies citrus sunburn fruits under complex orchard conditions, limitations remain due to the dataset’s limited variety and challenges in detecting severely occluded fruits. Future work will focus on expanding the dataset across varieties, stages, and environments, enhancing feature extraction, and integrating depth cameras to improve robustness. These efforts aim to support large-scale monitoring, disaster assessment, and early warning applications in smart orchard management.

### 4.2 Conclusions

In this study, citrus sunburn fruit in complex natural scenes was taken as the research object. Based on the YOLOv8n model, an identification and classification model for citrus sunburn named YOLOv8-Scm was constructed. Problems such as low detection precision and inaccurate recognition results are caused by the similarity in adjacent grading features, occlusion by branches and leaves, and inconsistent detection object sizes. To tackle these issues, this study created a dataset of citrus sunburn of diferent grades. Firstly, the Conv module was replaced with the DSConv module to reduce the quantity of model calculation parameters. Secondly, the GAM global attention mechanism was introduced to improve the precise monitoring capability of complex environment and citrus fruits of diferent sizes. Thirdly, we replaced the CIoU loss function with the EIoU loss function to enhance the model’s generalization capability, reduce errors, and mitigate over fitting during training. After comparing the YOLOv8-Scm model’s results with the state-of-the-art (SOTA) performance of mainstream identification modelsthe main conclusions are as follows:

Compared with the SSD, Faster R-CNN, YOLOv5n, YOLOv7-tiny, YOLOv8n, and YOLOv10n models, the mAP50 is increased by 6.01%, 2.87%, 6.5%, 5.3%, 2%, and 1.8%, respectively, and the precision P is increased by 7.1%, 13.4%, 3.8%, 4.5%, 1.5% and 1.9%, respectively. These findings clearly illustrate that the performance of the YOLOv8-Scm method is better than those of the above mainstream models and that its ability to detect the degree of damage to citrus sunburn fruit is feasible. In addition, the number of Params of this model is less than that of the other four versions of YOLOv8 (s, m, l, and x), and it is 23.77 M lower than the number of parameters of the newly released YOLOv10n network model. The reduction in the models weight is conductive to its deployment on the orchard panoramic camera platform, which is of practical significance in promoting smart orchard management.

## Data Availability

The raw data supporting the conclusions of this article will be made available by the authors, without undue reservation.
